# CiThroModel Improves Prediction of Symptomatic Venous Thromboembolism in Hospitalized Patients With Cirrhosis Without Hepatocellular Carcinoma

**DOI:** 10.1002/ueg2.12758

**Published:** 2025-01-23

**Authors:** Alberto Zanetto, Alessandro Vitale, Filippo Pelizzaro, Vittorio Simeon, Elena Campello, Laura Turco, Lorenz Balcar, Francesco Paolo Russo, Patrizia Burra, Paolo Simioni, Marco Senzolo

**Affiliations:** ^1^ Department of Surgery, Oncology and Gastroenterology University of Padova Padova Italy; ^2^ Gastroenterology and Multivisceral Transplant Unit Padova University Hospital Padova Italy; ^3^ Hepatobiliary Surgery and Liver Transplantation Unit Padova University Hospital Padova Italy; ^4^ Medical Statistics Unit University “Luigi Vanvitelli” Naples Italy; ^5^ Department of Medicine (DIMED) University of Padova Padova Italy; ^6^ First Chair of Internal Medicine and Thrombotic and Haemorrhagic Disease Unit Padova University Hospital Padova Italy; ^7^ Internal Medicine Unit for the Treatment of Severe Organ Failure IRCCS Azienda Ospedaliero‐Universitaria di Bologna Bologna Italy; ^8^ Division of Gastroenterology and Hepatology Internal Medicine III Medical University of Vienna Vienna Austria

**Keywords:** CiThroModel, heopatocellular carcinoma, liver cirrhosis, venous thromboembolism

## Abstract

**Background & Aims:**

Venous thromboembolism (VTE) is a recognized complication of acutely ill patients, but its incidence and risk factors in those with cirrhosis are uncertain.

**Methods:**

We retrospectively studied a consecutive cohort of cirrhosis patients non‐electively admitted to our medical unit to determine the rates of symptomatic VTE during hospitalization. Firstly, we explored associations with baseline, clinical and laboratory characteristics using logistic regression. Secondly, we developed a clinical prediction model that could predict the risk of in‐hospital VTE.

**Results:**

We included 687 patients (median age 61 years old; 68% male; Child–Pugh A/B/C, 13%/40%/47%). During hospitalization, 34 patients (4.9%) experienced VTE. Multivariate analysis showed that male sex (OR: 2.56, *p* = 0.05), AKI (OR: 3.1, *p* = 0.001), bacterial infections (OR: 2.6, *p* = 0.008), Pugh score (OR: 1.6. *p* < 0.001), family history of thrombosis (OR: 3.1, *p* = 0.04), reduced mobility (OR: 4.6, *p* < 0.001), and C‐reactive protein (OR: 1.1, *p* = 0.005) were independent predictors of VTE. We combined these variables in a prediction model (*Ci*rrhosis*Thro*mbosis*Model*) that accurately discriminated between high‐ and low‐risk patients. The AUROC of CiThroModel was significantly higher than that of Padua prediction score (0.882 vs. 0.742). After validating the CiThroModel using bootstrapping, the adjusted model maintained optimal discrimination ability (0.862) and calibration. The adjusted formula to calculate the in‐hospital risk of VTE was −9.00 + 0.82 [Male sex] + 1.14 [AKI] + 0.98 [Infection] + 0.48 * Child Pugh score + 1.14 [VTE family history] + 1.54 [Reduced mobility] + 0.15 * PCR/10.

**Conclusion:**

The CiThroModel seems a valuable tool for identifying hospitalized patients with cirrhosis at risk of VTE (https://majinzin.shinyapps.io/vterisk/).

## Graphical abstract

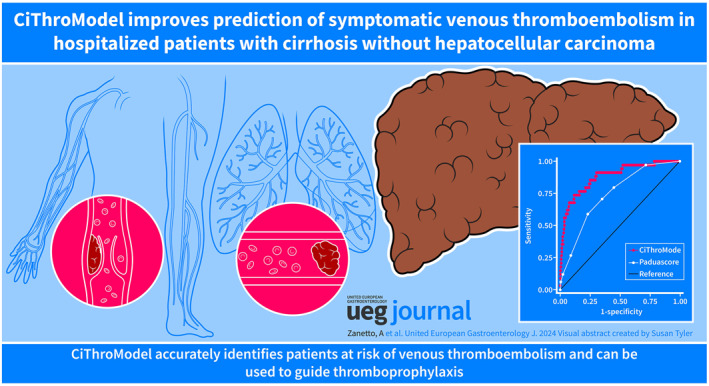

1

**Summary**

- Summarize the established knowledge on this subject:
  ◦ Venous thromboembolism (VTE) is a recognized complication of acutely ill patients, but its incidence and risk factors in those with cirrhosis are uncertain due to poor quality studies with confounding factors and non‐standardized definitions.
  ◦ Thromboprophylaxis in hospitalized patients with cirrhosis is still underused due to perceived risk of bleeding.
  ◦ A better definition of the thrombotic risk in hospitalized patients with cirrhosis may lead to a more individualized use of thromboprophylaxis.
- What are the significant and/or new findings of this study?
  ◦ Inpatients with cirrhosis are at risk of VTE, especially those with reduced mobility, severe liver dysfunction, bacterial infections, and acute kidney injury.
  ◦ Using a rigorous statistical methodology, we developed a new predictive model (CiThroModel) that accurately identifies patients at risk and can be used to guide thromboprophylaxis (https://majinzin.shinyapps.io/vterisk/).
  ◦ The following formula can be used to calculate the in‐hospital risk of VTE: −9.00 + 0.82 [Male sex] + 1.14 [AKI] + 0.98 [Infection] + 0.48 * Child Pugh score + 1.14 [VTE family history] + 1.54 [Reduced mobility] + 0.15 * PCR/10.

## 2 Introduction

Acutely ill patients are at risk of developing venous thromboembolism (VTE), with an estimated rate of 4%–15% [1]. Hospitalization is one of the main risk factors for VTE and accounts for up to 25% of all VTEs [2]. However, multicenter cohort studies examining the incidence of VTE and prophylactic trials evaluating the safety and efficacy of prophylactic anticoagulation have excluded patients with chronic liver diseases [3, 4, 5]. Retrospective studies have shown that the incidence of VTE in hospitalized patients with cirrhosis varies between 0.5% and 7% [6, 7]. A systematic review with meta‐analysis found that 1% of patients with cirrhosis developed VTE during hospitalization [8].

Caution is required in interpreting these trends since the higher risk of VTE in cirrhosis may reflect, at least partly, the under‐utilization of thromboprophylaxis due to perceived risk of bleeding [9, 10]. A retrospective, ICD codes‐based study including patients discharged from the Internal Medicine Departments of Spanish Public Health Service found that the prevalence of VTE in patients with moderate/severe liver disease, mild liver disease, and no liver disease was 0.9%, 2.4%, and 2.7%, respectively [11]. In a comparable, independent study from the United States, the risk of non‐splanchnic thrombosis in cirrhosis was slightly lower than in patients without liver disease [12].

Essentially, whether hospitalized patients with cirrhosis have a higher risk of VTE than inpatients without liver disease is unclear. Furthermore, the retrospective nature of most data with confounding factors and non‐standardized definitions makes it difficult to assess whether cirrhosis‐specific factors influence thrombotic risk and guide treatment‐decision [13].

A better definition of the thrombotic risk in hospitalized patients with cirrhosis may lead to a more individualized use of thromboprophylaxis, thereby reducing the risk of VTE and VTE‐driven morbidity and mortality [11, 14]. In this study, we aimed to develop a clinical prediction model that could improve thrombotic risk stratification and support decision‐making regarding thromboprophylaxis in hospitalized patients with cirrhosis. To achieve this aim, we first evaluated the prevalence and risk factors of VTE in a consecutive cohort of patients with cirrhosis admitted to our unit over a 5‐year period. Second, we developed and validated a clinical prediction model using a stepwise rigorous statistical methodology [15].

## 3 Methods

### 3.1 Patients and Study Design

This is a retrospective analysis of a prospective, single‐center cohort study wherein consecutive patients with cirrhosis admitted to the Gastroenterology and Multivisceral Transplant Unit of Padova University Hospital between January 1 2019 and February 28 2024 were screened for recruitment at the time of hospitalization (HIC protocol #0034435). The study was conducted in compliance with the principles of the Declaration of Helsinki, and all patients signed a consent to participate.

The diagnosis of cirrhosis was confirmed using available data, including histology, radiology, laboratory, and clinical assessment. Both compensated and decompensated patients were eligible for recruitment [16, 17].

Exclusion criteria were: acute‐on‐chronic liver failure (ACLF) at time of screening (defined according to the EASL‐CLIF consortium criteria as a clinical syndrome characterized by an intense systemic inflammatory response, single‐ or multiple organ system failures, and high 28‐day mortality) [18]; admission for variceal hemorrhage (VH) or experiencing VH and/or any major bleeding in the 30 days prior to admission [19]; admission to intensive care units; transfer from other hospitals; presence or history of portal vein thrombosis or VTE; chronic kidney disease; presence or history of any hepatic or extra‐hepatic tumor; known hematologic diseases or thrombophilic conditions; recent major surgery (within 1 month); HIV infection, history of any organ transplantation; anticoagulation and/or anti‐platelet therapy and/or anti‐fibrinolytic therapy; transfusion of red blood cells and/or fresh frozen plasma and/or platelet transfusions in the 7 days prior to screening.

All patients were followed during hospitalization until VTE, discharge, or death/liver transplantation, whichever came first. The time to VTE was calculated as the time (days) elapsed between patient recruitment and development of VTE. In patients who did not experience VTE, the duration of follow‐up was calculated between patient recruitment and discharge or death/liver transplantation.

In patients who experienced VTE, characteristics of thrombosis (i.e., isolated proximal and/or distal deep vein thrombosis [DVT] of the upper and/or lower limbs, DVT with pulmonary embolism [PE], or isolated PE) were collected. All VTEs were objectively proven by ultrasonography and/or CT scan. Diagnostic tests were applied if thrombotic complications were clinically suspected.

### 3.2 Data Collection

Data collected at the time of recruitment (i.e., hospital admission) included demographics, reasons for admission, laboratory data including conventional coagulation tests, C‐reactive protein (CRP), Model for End‐Stage Liver Disease (MELD) score, Child–Pugh stage, presence/absence of bacterial infections, presence/absence of acute kidney injury (AKI), presence/absence of reduced mobility, family history of venous thrombosis, and the Padua prediction score.

Bacterial infections were diagnosed and categorized as per standard criteria [20]. AKI was defined according to the International Club of Ascites as an increase in serum creatinine of greater than or equal to 0.3 mg/dL within 48 h or a 50% increase within 7 days from baseline serum creatinine [21]. Reduced mobility was defined as complete bed rest without bathroom privileges at the time of recruitment. The Padua prediction score is a clinical score that is, used to stratify thromboembolic risk in hospitalized medical patients and determine whether to initiate thromboprophylaxis [22].

### 3.3 Statistical Analysis

Qualitative data are described using frequency and percentage. Quantitative data are described using the median with 25% and 75% quartile ranges. Comparisons between independent groups were performed using the Mann Whitney U test for continuous variables and the Chi‐square test or Fisher's exact test for categorical variables. For comparison in three or more groups, the Kruskal–Wallis test was computed. Logistic regression was used to identify factors associated with the development of VTE.

To develop and validate our clinical prediction model, we strictly followed the methodology proposed by Strandberg et al. [15]. Briefly, after defining the need for a new prediction model (Step 1) as well as the purpose and intended use (Step 2) (please see introduction and study aim), we assessed the quality and quantity of our dataset (Step 3). Regarding quality, we checked that our database included all the potential predictors of VTE, including history (i.e., history of decompensation, etiology of liver disease, age, diabetes, etc.), clinical and physical examination (i.e., reduced mobility, signs of hepatic decompensation, etc.), laboratory data, and additional diagnostic and prognostic variables related to the severity of liver disease and associated complications (i.e., presence of infections, AKI, MELD score, etc.). Regarding quantity, we (A) decided to use a binary outcome instead of a time‐to‐event model because of the specific features of our dataset characterized by short‐term follow‐up with in‐hospital events [15, 23]; (B) estimated a minimum sample size (and maximum degree of freedom) to control the amount of overfitting in the prediction model. In Step 4, we developed the model using sound statistical methods, in particular: (A) we performed an advanced shrinkage technique (LASSO regression with penalized coefficient calculation) to reduce the model's overfitting; (B) we reduced the degree of freedom from 13 to 7 to fulfill the requirements calculated in Step 3; (C) we performed a logistic regression model. In Step 5, we used the model to generate VTE risk predictions between 0% and 100% on the probability scale. In step 6, (A) we evaluated the discrimination ability of the score by measuring the area under (AU) the receiver‐operating characteristic (ROC) curve, which was compared with the Padua prediction score [22], a score that is, currently recommended to stratify the risk of VTE in hospitalized patients with cirrhosis [6, 7]; (B) we performed two analyses to assess calibration (calibration belt and calibration plot); (C) we assessed the clinical utility of the model by a decision curve analysis. Finally, in Step 7, we validated the model using bootstrapping to correct for the apparent optimism in performance. A thorough description of each step and associated results can be found in Supporting Information S1.

For statistical analysis (AZ, AV, FP, and VS), IBM SPSS version 28 (Armonk, NY), R statistical software (version 4.3.0, R Core Team, Vienna, Austria), GraphPad Prism 8 (GraphPad Software, La Jolla, CA, USA), and STATA Stata/SE 18.0 (1985–2023) were used.

## 4 Results

### 4.1 Baseline Characteristics

Six‐hundred and eighty‐seven patients with cirrhosis (68% male, median age 61 years) were included (Figure S1). Alcohol and chronic viral hepatitis were the most common etiologies of cirrhosis (55% and 25%, respectively). Difficult to treat/recurrent ascites was the most common reason for admission (49%), followed by hepatic encephalopathy (17%), abdominal pain/suspected infection (14%), and AKI (8%) (Table 1). The median Pugh score was 9 (range 6–14); 13% of patients were Child–Pugh A, 40% were Child–Pugh B, and 47% of patients were Child–Pugh C.

**TABLE 1:** Baseline characteristics.

	Patients (*n* = 687)
Age, years	61 (54–68)
Male/female, %	69/31
Etiology of cirrhosis, %	
Alcohol	49
Alcohol‐MASH	6
HCV	17
HBV^b^	8
MASH	12
Others	8
Child‐Pugh A/B/C, %	13/40/47
Model for end‐stage liver disease (MELD) score	17 (12–24)
History of decompensation^c^, %	86
Reasons for admission, %	
Ascites grade ≥ 2	48
Hepatic encephalopathy	17
Abdominal pain/suspected infection	12
Acute kidney injury	7
Jaundice	2
Fatigue/anemia	7
Fall/trauma/fracture	5
Hyponatremia	2
Acute kidney injury^a^, %	30
Bacterial infections, %	27
Diabetes, %	20
Family history of thrombosis, %	5
Padua prediction score ≥ 4, %	25
Reduced mobility, %	35
Hemoglobin, g/dL	9 (8–12)
Platelet count, 10^9^/L	81 (56–119)
Thrombocytopenia, (%)	
Present	89
Mild > 100 ≤ 150 × 10^9^/L	25
Moderate 50 ≤ 100 × 10^9^/L	52
Severe < 50 × 10^9^/L	23
Total bilirubin, mg/dL	1.6 (0.8–3.4)
INR	1.4 (1.2–1.8)
Creatinine, mg/dL	0.8 (0.6–1.3)
Albumin, g/dL	31 (27–36)
C reactive protein, mg/L	14 (0.4–38)
Na, mmol/L	137 (134–139)
Patients receiving non‐selective beta blockers, %	42
Patients receiving diuretics, %	82
Patients receiving antibiotics, %	41
Patients receiving vasoactive drugs, %	18

*Note:* Median values reported with 25th and 75th percentile values in parenthesis.

^a^ 62/205 (30.2%) had serum creatinine ≥ 2 mg/dL.

^b^ Twenty‐one percent had HDV co‐infection.

^c^ Eighty‐two percent of patients with history of decompensation had ascites grade ≥ 2 at time of recruitment.

The median level of CRP was 14 mg/L. Approximately all patients were thrombocytopenic with the majority having moderate thrombocytopenia. Median INR was 1.4 (1.2–1.8) (Table 1). Thirty‐three patients (4.8%) had a family history of VTE; the Padua prediction score was ≥ 4 in 173 patients (25.2%).

*Hospitalized patients with cirrhosis are at risk of VTE, especially those with reduced mobility, advanced liver dysfunction, bacterial infections, and AKI.*

The median (range) duration of admission was 7 (3–45) days. During admission, 34 patients (4.9%) developed symptomatic VTE: 26 (76.6%) had DVT, 6 had PE (17.6%), and two isolated PE (5.8%). Among patients with isolated DVT, this was proximal in 17 patients and distal in 9 patients. The median time from recruitment to development of VTE was 8 days (range: 4–15). All patients who experienced VTE were treated with anticoagulation and none of these patients died due to VTE‐related causes. Fifty‐two patients died during index hospitalization due to non‐VTE related causes. In these patients, the median time from admission to death was 12 days (5–45).

Compared to patients who did not develop VTE, those with VTE had significantly higher MELD score and Child class (Table S1). Bacterial infections, AKI, and reduced mobility were all significantly more common in patients who experienced VTE versus those who did not. Among patients who experienced VTE, 97% were thrombocytopenic (33% having a platelet count < 50 × 10^9^/L). A severe coagulopathy (i.e., platelet count < 50 × 10^9^/L and INR > 2) was significantly more common in patients who developed VTE (Table S1).

Univariate analysis showed that alcohol‐related etiology, AKI, bacterial infections, presence of ascites, MELD score, Child–Pugh score, family history of thrombosis, reduced mobility, severe coagulopathy, hemoglobin, CRP, INR, bilirubin, Na, and Padua score ≥ 4 were associated with the development of VTE (Table S2).

*The CiThroModel identifies patients with cirrhosis at risk of symptomatic VTE better than the Padua prediction score.*

We performed an advanced shrinkage technique (LASSO regression with penalized coefficient calculation) to prevent the model from overfitting and select the variables of interest. Among the 13 variables initially identified (sex, alcohol‐related etiology, AKI, bacterial infections, hepatic encephalopathy, Child–Pugh score, family history of thrombosis, reduced mobility, severe coagulopathy, C‐reactive protein, creatinine, Na+), 7 were finally included in the multivariate model (Table 2). Reduction from 12 to 7 was required to fulfill the requirements as per the maximum degree of freedom based on the number of patients included in our study (See Supporting Information S1 Step #3 and #4 for additional details regarding LASSO analysis, selection of study variables, and final logistic model).

**TABLE 2:** Multivariate model.

Variable	Odds ratio	Std. err.	*Z*	*p* > |*z*|	95% CI
Original					
Male sex	2.563	1.260	1.92	0.055	0.978–6.717
AKI (acute kidney injury)	3.689	1.150	3.21	0.001	1.661–8.190
Bacterial infections	3.065	1.289	2.66	0.008	1.343–6.993
Child‐Pugh score	1.742	0.230	4.20	0.000	1.344–2.258
Family history of thrombosis	3.683	2.344	2.05	0.040	1.058–12.824
Reduced mobility	5.830	2.541	4.05	0.000	2.481–13.698
C‐reactive protein/10	1.186	0.071	2.83	0.005	1.053–1.335
Adjusted by bootstrap shrinkage					
Male sex	2.270	0.971	1.92	0.055	0.981–5.254
AKI (acute kidney injury)	3.117	1.104	3.21	0.001	1.556–6.244
Bacterial infections	2.653	0.972	2.66	0.008	1.293–5.441
Child‐Pugh score	1.622	0.186	4.20	0.000	1.294–2.033
Family history of thrombosis	3.113	1.725	2.05	0.040	1.050–9.227
Reduced mobility	4.644	1.762	4.05	0.000	2.207–9.773
C‐reactive protein/10	1.160	0.061	2.83	0.005	1.046–1.286

**FIGURE 1:**
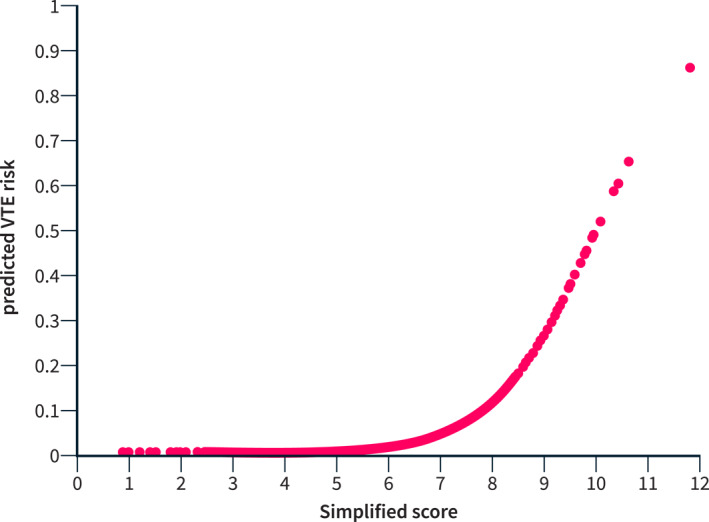
Predicted risk of VTE. Each individual predicted risk of VTE was plotted over the simplified score (E + 10) derived from our final equation (E). VTE, venous thromboembolism.

The model was used to generate the VTE risk predictions. By inputting the predictors into the logistic function, we obtained a value between 0 and 1, corresponding to a risk scale between 0% and 100%. The final equation (E) was: = −10.03 + 0.94 [Male sex] + 1.31 [AKI] + 1.12 [bacterial infection] + 0.56 * Child‐Pugh score + 1.30 [VTE family history] + 1.76 [Reduced mobility] + 0.17 × PCR/10. The brackets “[ ]” are equal to 1 if the patient has a specific characteristic (i.e., if the patient is male, has AKI, has a bacterial infection, has VTE family history, has reduced mobility), and 0 otherwise. The predicted risk of in‐hospital VTE was calculated as follows: 1/(1 + Exp (−E)), where Exp is equal to 271,828,182,845,904 (i.e., Nepero number). Figure 1 shows a simplified version of the score (E + 10) to visualize the increasing risk of VTE.

**FIGURE 2:**
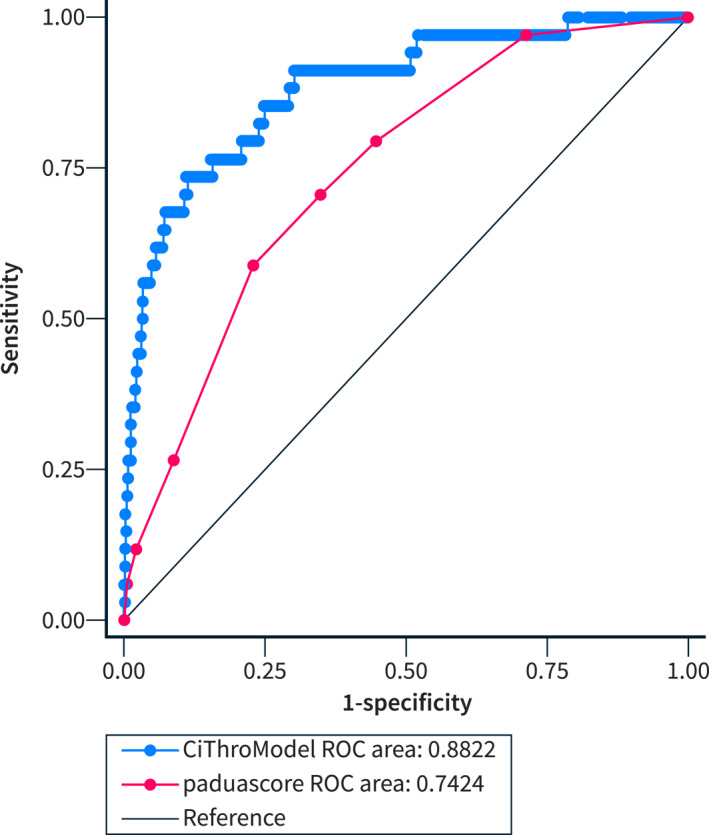
The AUROC of the CiTrhoModel was significantly higher than that of the Padua prediction score. Delta AUROC was 14% (*p* < 0.001).

**FIGURE 3:**
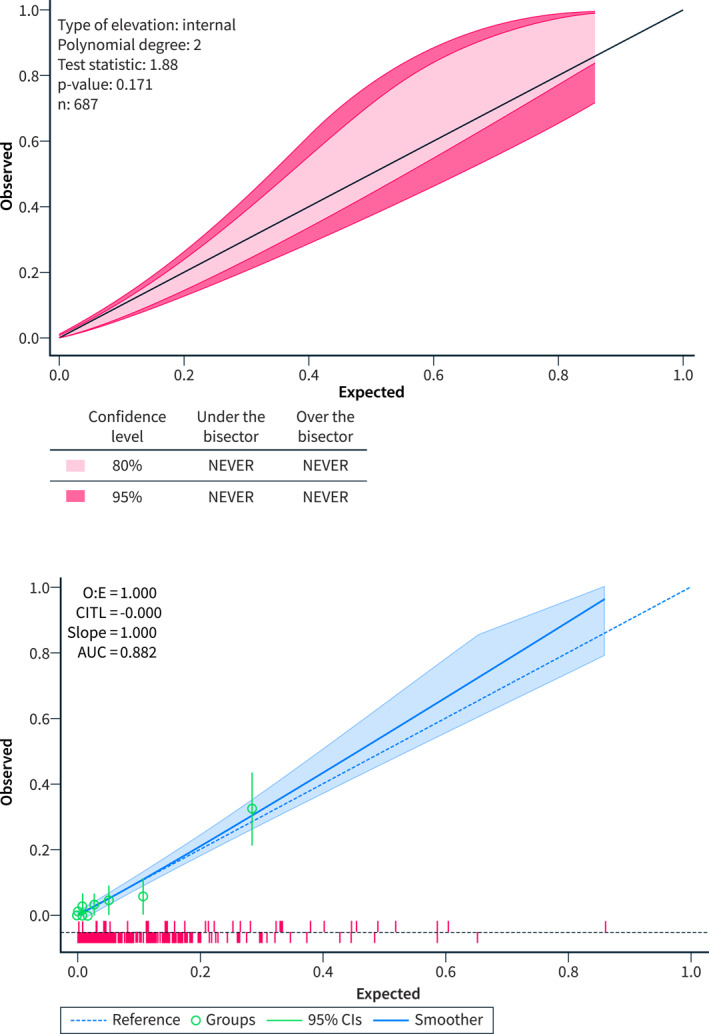
The calibration belt (left) and calibration curve (right) showed that the CiTrhoModel had acceptable internal calibration.

The area under (AU) the receiver‐operating characteristic (ROC) curve was 0.8822, indicating an excellent discrimination of CiThroModel. Notably, the CiThroModel's AUROC was significantly higher than that of the Padua prediction score (Figure 2). Calibration belt and calibration plot analyses showed a good calibration of the model (Figure 3).

**FIGURE 4:**
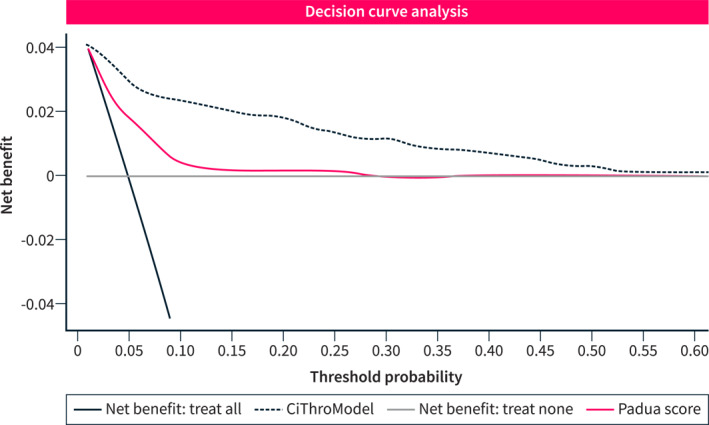
Decision curve analysis. The decision curve analysis shows that the CiThroModel had a higher net benefit (i.e., the relative benefits and harms of VTE prophylaxis at different thresholds of predicted VTE probability) compared to the Padua prediction score.

Figure 4 shows the net benefit analysis (i.e., decision curve). This analysis highlights the clear superiority of the CiThroModel compared to the Padua score in terms of net benefit (i.e., the relative benefits and harms of VTE prophylaxis at different thresholds of predicted VTE probability). For instance, at a given threshold probability of 0.10, the figure shows a difference in net benefit of 0.020 between the CiThroModel and the Padua scores. This difference could be interpreted as “using the CiThroModel instead of the Padua score to decide VTE prophylaxis increases the number of VTE detected (true positives) by 20 per 1000 patients, without changing the number of unnecessary treatments (false positives).”

**FIGURE 5:**
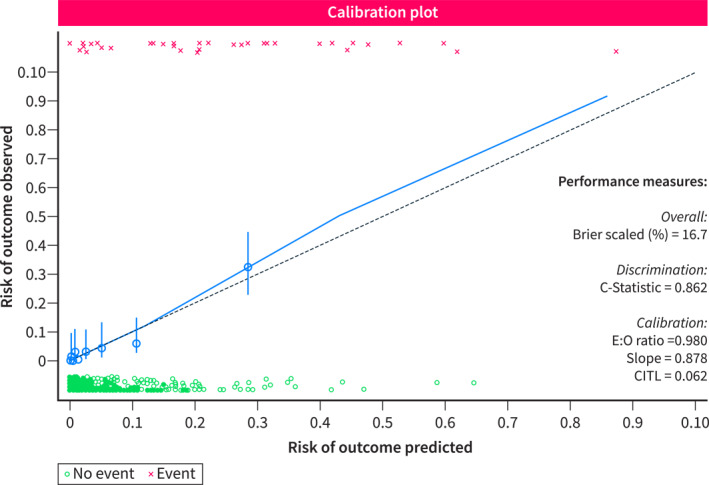
Adjusted calibration plot after bootstrapping.

Finally, we validated the model using bootstrapping to correct for the apparent optimism in performance (Table 2). After bootstrapping, the adjusted model maintained an optimal discrimination ability (C‐Statistic of 0.862) and calibration (E:O ratio = 0.980, CITL = 0.062, Slope = 0.878). The adjusted calibration plot is shown in Figure 5. Based on the new coefficients, the adjusted (final) CiThroModel was E = −9.00 + 0.82 [Male sex] + 1.14 [AKI] + 0.98 [Infection] + 0.48 * Child‐Pugh score + 1.14 [VTE family history] + 1.54 [Reduced mobility] + 0.15 × PCR/10.

## 5 Discussion

The risk of VTE in hospitalized patients with cirrhosis is uncertain because of low‐quality data from retrospective series, including heterogenous cohorts with multiple confounding factors [6]. A better understanding of the thrombotic risk associated with cirrhosis would lead to a more rational use of thromboprophylaxis and potentially mitigate VTE‐driven morbidity and mortality [6, 7].

Our study shows, in a consecutive cohort of hospitalized patients with cirrhosis, that the overall rate of in‐hospital symptomatic VTE was 4.9%. Previous retrospective studies showed that the prevalence of VTE in cirrhosis ranges between 1% and 7% [14, 24]. These wide ranges reflect the inclusion of patients at different stages of cirrhosis (compensated vs. decompensated vs. ACLF), with variable comorbidities and/or risk factors for VTE, receiving different prophylactic strategies (anticoagulation vs. no anticoagulation vs. variable doses/duration of prophylaxis) [6]. In our study, we included prospectively characterized patients who were not receiving anticoagulation. Although our exclusion criteria partly limited the assessment of other potential risk factors for VTE (i.e., chronic kidney disease, heart/respiratory failure, and liver cancer), we were able to recruit a homogenous cohort and clearly identify patients at high versus low risk, which is key when evaluating the *individual* need for thromboprophylaxis.

Anticoagulant prophylaxis is under‐prescribed in patients with cirrhosis because of the perceived risk of bleeding [25]. However, a prolonged INR and/or a low platelet count does not confer any protection against VTE [26, 27]. In our cohort, 99% of patients who developed VTE were thrombocytopenic, with 33% having a platelet count < 50 × 10^9^/L. Moreover, patients who experienced VTE had a significantly more severe coagulopathy than those who did not.

Alterations in conventional coagulation tests should not be considered as a contraindication to anticoagulation. Instead, they indicate a more severe liver disease, which was associated with VTE [27]. The association between Child–Pugh C and VTE likely reflects the increasing severity of portal hypertension, bacterial translocation, and systemic inflammation, which would drive a stronger activation of coagulation and increase thrombotic risk [28]. We found that bacterial infections were associated with VTE, thus confirming previous studies in medical [29] and critically ill [30] patients with chronic liver disease. Infections and sepsis in decompensated cirrhosis have been associated with pro‐thrombotic alterations such as a higher level of FVIII and reduced anticoagulants [13, 20]. Interestingly, we first reported that AKI was also associated with VTE, which confirmed the recent hypothesis that hyper‐coagulable changes in AKI‐cirrhosis may be responsible for thrombosis [31].

Current guidelines suggest using the Padua prediction score to estimate the thrombotic risk in hospitalized patients with cirrhosis. Although the original study did not include patients with chronic liver disease [22], it was later shown that the Padua score can be used to identify cirrhosis patients at risk of VTE [32]. However, the Padua score lacks specific metrics of liver disease severity and common complications of cirrhosis such as infections and AKI [24, 27]; moreover, many of the items included in the Padua prediction score such as acute myocardial infarction/stroke, rheumatologic disorders, known thrombophilic condition, or hormonal therapy are less common/applicable in cirrhosis than in general medical patients.

The European Association for the Study of the Liver (EASL) and International Society for Thrombosis and Hemostasis (ISTH) guidelines state that observational studies are needed to test the real‐life ability of clinical prediction scores to assess the risk for VTE in cirrhosis [6, 7]. Therefore, we created a simple predictive model (i.e., CiThroModel) that accurately identified the subset of patients at risk of symptomatic VTE (https://majinzin.shinyapps.io/vterisk/). We followed a rigorous statistical methodology which has been recently proposed for the development of a clinical prediction model in hepatology [15]. Remarkably, the prognostic performance of our model was significantly higher than that of the Padua prognostic score (AUROC 0.882 vs. 0.742, respectively), suggesting that the assessment of cirrhosis‐specific risk factors may improve risk stratification. Another potential explanation for the lower performance of Padua prediction score compared with CiThroModel is that we excluded patients with cancers, which is a well‐known major risk factor for thrombosis. It should be highlighted that we excluded patients with cancer. Therefore, the comparison with the Padua prediction score may be partly biased, necessitating further studies that include patients with cancer to evaluate whether the presence of HCC or other liver tumors increases the risk of in‐hospital VTE in acutely decompensated cirrhosis. However, this allowed us to better assess the pro‐thrombotic role of cirrhosis‐specific factors such as decompensation severity, AKI, and infections. By following our decision curve analysis, one could easily distinguish high‐risk patients (i.e., anticoagulant prophylaxis is largely justified) [7] versus low‐risk patients (i.e., anticoagulation might not be necessary). Importantly, our model's prognostic performance and calibration were confirmed after correcting for the apparent optimism in performance by a bootstrap analysis.

Our study has significant limitations. Firstly, since hospitalized patients with cirrhosis are in a dynamic condition that may vary, a repeated versus baseline assessment of thrombotic risk may improve the identification of patients at risk [33]. Notably, patients were screened and recruited at hospital admission. Therefore, it is possible that some of these patients were later started on thromboprophylaxis during admission. Systemic inflammation and development of ACLF have been linked to an increased risk of thrombosis in acutely decompensated cirrhosis [34]. Thus, in clinical practice, a dynamic assessment of the patient's condition is likely the key to define thrombo‐hemorrahgic risk. Secondly, these results from a single‐center study need external validation. Before the CiThroModel can be implemented in clinical practice. While the unique characteristics of our cohort allowed for a detailed examination of predisposing factors for VTE risk in cirrhosis, we were unable to include an external validation cohort with comparable numbers and characteristics. Although the ideal statistical approach for predictive modeling involves both internal and external validation, we have made efforts to address this issue. In fact, we performed a bootstrap analysis, which is currently the statistical gold standard in the absence of an independent validation cohort [15]. Thirdly, we aimed to develop a prediction model based on standard clinical and laboratory data; whether specific coagulation tests could improve the prediction of VTE in cirrhosis should be further explored [35]. Finally, we employed several exclusion criteria to ensure a homogenous population for assessing thrombotic risk factors. However, this does limit the clinical applicability of the CiThroModel, and validation in more diverse populations is essential. It is important to note that patients with ACLF and those with recent variceal hemorrhage were not eligible for recruitment in the current cohort. Therefore, the applicability of our findings to these individuals remains uncertain and warrants further investigation.

In conclusion, hospitalized patients with cirrhosis without hepatocellular carcinoma are at risk of VTE. The CiThroModel (−9.00 + 0.82 [Male sex] + 1.14 [AKI] + 0.98 [Infection] + 0.48 * Child Pugh score + 1.14 [VTE family history] + 1.54 [Reduced mobility] + 0.15 × PCR/10) can be used to identify the patients at higher risk of thrombosis and guide thromboprophylaxis.

## Author Contributions

A.Z.: research idea and design, recruitment of the patients, collection and interpretation of the data, statistical analysis, and writing of the manuscript. A.V.: interpretation of the data, statistical analysis (development of the model). Revision of the manuscript, and final approval. F.P.: statistical analysis and revision of the manuscript. V.S.: statistical analysis. E.C.: interpretation of the data and revision of the manuscript. L.T.: revision of the manuscript. L.B.: revision of the manuscript. F.P.R.: acquisition of the data and revision of the manuscript. P.B.: acquisition of the data and revision of the manuscript. P.S.: research idea and design, interpretation of the data, revision of the manuscript, and final approval. M.S.: research idea and design, acquisition and interpretation of the data, revision of the manuscript. And final approval.

## Ethics Statement

This study was approved by the Padova University Hospital (HIC protocol #0034435‐08/06/20) and was conducted in compliance with the principles of the Declaration of Helsinki. All patients signed a consent to participate.

## Conflicts of Interest

The authors declare no conflicts of interest.

## Supporting information

Supporting Information S1

## Data Availability

Data are available from the first author (Alberto Zanetto; email: alberto.zanetto@unipd.it) upon reasonable request.
