# Supplementary material for: CiThroModel Improves Prediction of Symptomatic Venous Thromboembolism in Hospitalized Patients With Cirrhosis Without Hepatocellular Carcinoma
Source: United European Gastroenterol J. 2025 Jan 23;13(5):728–37. doi: 10.1002/ueg2.12758 (PMC12188346; doi:10.1002/ueg2.12758)
Supplement: Supplementary file 1 — Supporting Information S1 [file UEG2-13-728-s001.docx]

**Title**: CiThroModel improves prediction of symptomatic venous thromboembolism in hospitalized patients with cirrhosis.

**Author names**: Alberto Zanetto^1,2^, Alessandro Vitale^1,3^, Filippo Pelizzaro^1,2^, Vittorio Simeon^4^, Elena Campello^5,6^, Laura Turco^7^, Lorenz Balcar^8^, Francesco Paolo Russo^1,2^, Patrizia Burra^1,2^, Paolo Simioni^5,6*^, Marco Senzolo^1,2*^

*Co-senior authors.

**Table of contents**

**Supporting tables………………………………………………………………………………...2**

**Supporting figures……………………………………………………………………………….5**

**Development of the CiThroModel………………………………………………………………6**

**Supporting references…………………………………………………………………………..20**

**Supporting Table 1. Comparison between baseline characteristics in cirrhosis patients who experienced VTE during hospitalization vs. those who did not.**

|  | **VTE**  (n=34) | **No VTE**  (n=653) | **p** |
| --- | --- | --- | --- |
| Age, years | 62 (47-65) | 61 (54-68) | 0.2 |
| Male, % | 79 | 67 | 0.2 |
| MELD score | 24 (20-30) | 17 (12-23) | <0.001 |
| Child A/B/C, % | 0/18/82 | 14/41/45 | <0.001 |
| Bacterial infection, % | 61 | 25 | <0.001 |
| Acute kidney injury, % | 62 | 28 | <0.001 |
| Diabetes, % | 11 | 25 | 0.1 |
| Hemoglobin, g/dL | 8.6 (7.8-10.2) | 9.6 (8.1-11.9) | 0.02 |
| Creatinine, mmol/L | 1.1 (0.7-1.8) | 0.8 (0.7-1.3) | 0.09 |
| Bilirubin, mg/dL | 5.6 (2.6-10.1) | 2.4 (1.2-5.2) | <0.001 |
| Albumin, g/L | 29.5 (26-33) | 31 (27-36) | 0.04 |
| Platelet count, x10^9^/L | 73 (48-119) | 81 (57-122) | 0.2 |
| Thrombocytopenia, % | 97 | 83 | 0.1 |
| INR | 1.8 (1.5-2.2) | 1.5 (1.3-1.8) | <0.001 |
| Severe coagulopathy (platelet count <50x10^9^/L and INR >2), % | 15 | 5 | 0.009 |
| C-reactive protein, mg/dL | 28 (12-48) | 9 (3-28) | <0.001 |
| Family history of thrombosis, % | 14 | 4 | 0.006 |
| Padua prediction score ≥4, % | 64 | 23 | <0.001 |
| Reduced mobility, % | 67 | 33 | <0.001 |
| Age >70 years | 4 | 17 | 0.07 |

Median values are reported, with 25th and 75th percentile values in parenthesis. Legend: MELD, Model for End-Stage Liver Disease.

**Supporting Table 2. Parameters associated with VTE at univariate logistic regression analyses.**

| **Variable** | **Univariable** | |
| --- | --- | --- |
|  | **OR (95% CI)** | **p** |
| Age | 0.972 (0.943-1.003) | 0.07 |
| Sex  Female  Male | **-**  1.815 (0.778-4.236) | -  0.16 |
| **MELD score** | **1.11 (1.064-1.166)** | **<0.001** |
| **Child-Pugh score** | **1.71 (1.376-2.144)** | **<0.001** |
| **Ascites**  **No**  **Yes** | **-**  **6.372 (1.078-5.104)** | **-**  **0.03** |
| Hepatic encephalopathy  No  Yes | -  1.487 (0.736-3.002) | -  0.26 |
| **Family history of thrombosis**  **No**  **Yes** | **-**  **3.848 (1.385-10.692)** | **-**  **0.01** |
| **Alcohol-related liver disease**  **No**  **Yes** | **-**  **2.346 (1.078-5.104)** | **-**  **0.03** |
| Virus-related liver disease  No  Yes | -  0.785 (0.335-1.838) | -  0.57 |
| MASLD-related liver disease  No  Yes | -  0.202 (0.027-1.499) | -  0.11 |
| Diabetes  No  Yes | -  0.378 (0.113-1.256) | -  0.11 |
| Platelet count, x10^9^/L | 0.994 (0.987-1.002) | 0.18 |
| **INR** | **2.234 (1.334-3.737)** | **0.002** |
| **Bilirubin, mg/dL** | **1.057 (1.014-1.101)** | **0.008** |
| Albumin, g/dL | 0.972 (0.929-1.017) | 0.22 |
| Creatinine, mg/dL | 1.300 (0.897-1.882) | 0.16 |
| **Na, mEq/L** | **0.923 (0.863-0.986)** | **0.01** |
| **Severe coagulopathy**  **No**  **Yes** | **-**  **3.848 (1.385-10.692)** | **-**  **0.01** |
| **Hemoglobin, g/dL** | **0.805 (0.673-0.963)** | **0.02** |
| **CRP, mg/L (increase of 10 mg/L)** | **1.162 (1.063-1.270)** | **0.001** |
| **AKI**  **No**  **Yes** | **-**  **4.05 (1.989-8.268)** | **-**  **<0.001** |
| **Bacterial infection**  **No**  **Yes** | **-**  **4.896 (2.397-9.999)** | **-**  **<0.001** |
| **Reduced mobility**  **No**  **Yes** | **-**  **4.201 (2.010-8.776)** | **-**  **<0.001** |
| **Padua score ≥4**  **No**  **Yes** | **-**  **4.790 (2.362-9.713)** | **-**  **<0.001** |

**Supporting Figure 1. Flow chart of the study.**

Abbreviations: ACLF: acute-on-chronic liver failure; VH: variceal hemorrhage; ICU: intensive care unit; PVT: portal vein thrombosis; VTE: venous thromboembolism.

*******DEVELOPING AND VALIDATING THE CiThroModel*******

*Below, we will describe the nine steps to developing and validating the Cirrhosis Thrombosis Model (CiThroModel), following Strandberg R et al.'s methodology (1).

***1. ****DETERMINE IF THERE IS A NEED FOR A NEW PREDICTION MODEL******

***2 ****FORMULATE THE PROBLEM AND DEFINE THE INTENDED USE******

*Multicenter cohort studies examining the incidence of VTE and prophylactic trials evaluating the safety and efficacy of prophylactic anticoagulation have excluded patients with chronic liver diseases.

* There are two main problems: 1. A better understanding of VTE incidence and risk factors in hospitalized patients with cirrhosis is required. 2. There is an unmet need for better risk stratification regarding the use of thromboprophylaxis in hospitalized patients with decompensated cirrhosis. In fact, anticoagulant prophylaxis is still under-prescribed in these patients due to a perceived risk of bleeding associated with low platelet count and/or prolonged INR.

Therefore, a model predicting the risk of VTE in hospitalized cirrhotic patients would improve patient management. Accurately predicting this risk is the only way to offer an objective and effective clinical tool for deciding when to prescribe anticoagulation prophylaxis in these patients.

***3***ASSESS THE QUALITY AND QUANTITY OF THE DATA YOU WISH TO DEVELOP THE MODEL ON******

*****QUALITATIVE ASSESSMENT*****

*We analyzed a database characterized by a consecutive cohort of patients with cirrhosis non-electively admitted to our medical unit. The database included 687 patients (median age 61; 68% male; Child-Pugh A/B/C,13%/40%/47%). We performed a qualitative assessment proving that the database included all the potential predictors of interest as possible risk factors of VTE: anamnestic (age, sex, history of previous VTE, history of liver decompensation, diabetes, etiology of liver disease), clinical/including a physical examination (presence/absence of reduced mobility, signs of liver decompensation such as hepatic encephalopathy, ascites), laboratory data (conventional coagulation tests, C-reactive protein (CRP), hemoglobin, platelet count, bilirubin, creatinine, albumin, sodium), complex diagnostic/prognostic variables (presence reasons for admission, presence of bacterial infections, acute kidney injury (AKI), severe coagulopathy, Model for End-Stage Liver Disease (MELD) score, Child-Pugh score).

*****QUANTITATIVE ASSESSMENT*****

****Selection of the prediction model****

*We decided to use a BINARY OUTCOME instead of a time-to-event model due to the specific setting of our study characterized by short term "in-hospital" events (1-5).

A total of 52 patients (7.6%) who died due to non-VTE related causes during hospitalization were considered as controls, as were regularly discharged patients. This appeared to be an acceptable assumption for the model after observing the median time to events of the included patients:

*Median time to discharge = 7 days (range, 3-45).

*Median time to death (non VTE related) = 12 days (range, 5-45).

*Median time to VTE = 8 days (range, 4-15).

****Estimation of the minimum sample size (or maximum degrees of freedom)****

*pmsampsize, type(b) cstatistic(0.86) parameters(10) prevalence(0.049)*

*pmsampsize, type(b) cstatistic(0.86) n(687) prevalence(0.049)*

*help pmsampsize*

Given C-statistic = .86 & prevalence = .049

Cox-Snell R-sq = 0.0964

NB: Assuming 0.05 acceptable difference in apparent & adjusted R-squared

NB: Events per Predictor Parameter (EPP) assumes prevalence = .049

Sample size Shrinkage Parameter CS_Rsq Max_Rsq Nag_Rsq EPP

-------------+--------------------------------------------------------------------------------------------------

Criteria 1 | 687 .9 7 .0964 .324 .298 4.81

Criteria 2 | 687 .856 11 .0964 .324 .298 3.06

Criteria 3 * | 687 .9 7 .0964 .324 .298 4.81

-------------+-------------------------------------------------------------------------------------------------

Final | 687 .9 7 .0964 .324 .298 4.81

*Maximum number of predictor parameters that could be estimated during new model development based on user inputs = 7, with 34 events (assuming an outcome prevalence = .049) & an EPP = 4.81

* 95% CI for overall risk = (.033, .065), for true value of .049, sample size n=687

*Absolute margin of error = .016

Criteria 1 - small overfitting defined as expected shrinkage of predictor effects by 10% or less

Criteria 2 - small absolute difference in the model's apparent and adjusted Nagelkerke's R-squared

Criteria 3 - precise estimation of the average outcome risk in the population

***4. ****DEVELOP THE MODEL USING SOUND STATISTICAL METHODS******

*The sample size and degrees of freedom calculations made in Step 3 are intended to control the amount of overfitting in the prediction model. We performed an advanced shrinkage technique to reduce the model's overfitting (LASSO regression with penalized coefficient calculation). LASSO LOGIT regression was also crucial for the selection of variables.

*VTE prediction model

*LASSO LOGIT group, age, sex, virus, alcohol, MASLD, AKI, bacterial infections, ascites, hepatic encephalopathy, decomposed cirrhosis, Child class, Child-Pugh score, MELD, diabetes, family history of thrombosis, reduced mobility, severe coagulopathy, hemoglobin, platelet count, C-reactive protein, INR, bilirubin, albumin, creatinine, Na+, selection (adaptive)*

*LASSO coef., display (coef., penalized)*

Lasso logit model No. of obs. = 687

No. of covariates = 25

Selection: Adaptive No. of lasso steps = 2

Final adaptive step results

-----------------------------------------------------------------------------------------------------------------

| No. of Out-of-

| nonzero sample CV mean

ID | Description lambda coef. dev. ratio deviance

---------+------------------------------------------------------------------------------------------------------

33 | first lambda 1.827975 0 -0.0050 .3960018

79 | lambda before .0253154 12 0.2413 .2989538

*80 | selected lambda .0230664 12 0.2414 .2989109

81 | lambda after .0210173 13 0.2413 .298948

125 | last lambda . 0002652 16 0.1823 .3221866

-----------------------------------------------------------------------------------------------------------------

* Lambda selected by cross-validation in final adaptive step.

. LASSO coef., display(coef., penalized)

----------------------------------------------------------

| active

-------------+-------------------------------------------

Male sex | .7927105

Alcohol | .5127658

AKI | 1.510099

Bacterial infection | 1.07067

Hepatic encephalopathy | -.4468962

Child-Pugh score | .5376362

Family history of thrombosis | 1.15654

Reduced mobility | 1.729145

Severe coagulopathy | .4324429

C-reactive protein | .0162287

Creatinine | -.1812705

Na+ | -.0536487

_cons | -4.305326

-------------------------------------------------------

Legend:

b - base level

e - empty cell

o – omitted

***Full logistic model. We used PCR/10 to increase the readability of this variable.**

*Logistic variable, sex, alcohol, AKI, bacterial infection, hepatic encephalopathy, Child-Pugh score, diabetes, family history of thrombosis, reduced mobility, severe coagulopathy, C-reactive protein/10, creatinine, Na+.*

Logistic regression Number of obs = 687

LR chi2(13) = 94.15

Prob > chi2 = 0.0000

Log likelihood = -88.274232 Pseudo R2 = 0.3478

------------------------------------------------------------------------------------------------------------------

Variable | Odds ratio Std. err. z P>|z| [95% conf. interval]

-------------+---------------------------------------------------------------------------------------------------

Sex | 2.628811 1.358093 1.87 0.061 .9550206 7.236126

Alcohol | 2.051659 .9728842 1.52 0.130 .8099733 5.196845

AKI | 6.809799 3.824111 3.42 0.001 2.265341 20.47081

Bacterial infection | 3.189151 1.39888 2.64 0.008 1.349915 7.534315

Hepatic encephalopathy | .5030059 .2210628 -1.56 0.118 .2125612 1.190315

Child-Pugh score | 1.772773 .2565038 3.96 0.000 1.335033 2.354041

Family History of thrombosis | 3.507198 2.289999 1.92 0.055 .9753816 12.6109

Mobility | 6.450573 2.941944 4.09 0.000 2.63868 15.76921

Severe coagulopathy | 1.879131 1.246718 0.95 0.342 .5119454 6.89748

CRP/10 | 1.192729 .0758807 2.77 0.006 1.052904 1.351123

Creatinine | .6144333 .2250201 -1.33 0.184 .2997398 1.25952

Na | .9323507 .0340411 -1.92 0.055 .8679626 1.001515

_cons | .0667785 .3431868 -0.53 0.598 2.82e-06 1581.664

-----------------------------------------------------------------------------------------------------------------

Note: _cons estimate baseline odds.

*At this point, we aimed to reduce the degrees of freedom from 13 (full model above) to 7 (to fulfil the requirements as per the Step 3). Therefore, we plotted each predictor's chi-squared (𝜒2) statistics, which reflects how accurately the predictor explains the data (and is used for calculating the predictor's p-value). If the predictor is truly uninformative, the expected value of the statistic equals the number of degrees of freedom used. If we subtract the degrees of freedom from each statistic, they all have an expected value of 0. We can then rank the predictors and reduce the degrees of freedom used for the less informative predictors.

The relatively less informative variables we removed corresponded to variables already somehow included in the complex variables (i.e., hepatic encephalopathy, severe coagulopathy, creatinine), or variables that we judged less relevant based on their clinical and pathophysiological relevance in this specific field (i.e., Sodium, Alcohol). We also evaluated the relationship between the outcome and continuous variables (Child-Pugh score and PCR). We found that the predictive performance of these variables was better explained by a linear than a non-linear (i.e., restricted cubic spline) relationship.

. logistic group sex alcohol aki infection he childscore history mobility severecoag pcr10 creat na

| **Variable** | **Estimate** | **Standard error** | **Chi-square** | **Prob>ChiSquare** |
| --- | --- | --- | --- | --- |
| Intercept | 1,2065964 | 5,2151119 | 0,05 | 0,8170 |
| Male sex | -0,4832659 | 0,2583094 | 3,50 | 0,0614 |
| AKI [0] | -0,9591813 | 0,28078 | 11,67 | 0,0006* |
| Bacterial infection [0] | -0,5798773 | 0,2193186 | 6,99 | 0,0082* |
| Pugh score | 0,57254476 | 0,1446908 | 15,66 | <,0001* |
| Family history of thrombosis [0] | -0,6274087 | 0,3264713 | 3,69 | 0,0546 |
| Reduced mobility [0] | -0,9320845 | 0,2280374 | 16,71 | <,0001* |
| Na | -0,0700463 | 0,0365111 | 3,68 | 0,0550 |
| CRP/10 | 0,17624402 | 0,0636194 | 7,67 | 0,0056* |
| Alcohol [0] | -0,3593244 | 0,2370969 | 2,30 | 0,1296 |
| Hepatic encephalopathy [0] | 0,34357674 | 0,2197418 | 2,44 | 0,1179 |
| Severe coagulopathy [0] | -0,3154047 | 0,3317272 | 0,90 | 0,3417 |
| Creatinine | -0,487055 | 0,3662238 | 1,77 | 0,1835 |

***Final logistic model**

*Logistic group sex, AKI, bacterial infections, Child-Pugh score, history of thrombosis, reduced mobility, CRP/10*

Logistic regression Number of obs = 687

LR chi2(7) = 81.87

Prob > chi2 = 0.0000

Log likelihood = -94.410065 Pseudo R2 = 0.3025

---------------------------------------------------------------------------------------------------------------------

Variable | Odds ratio Std. err. z P>|z| [95% conf. interval]

-------------+------------------------------------------------------------------------------------------------------

Sex | 2.563651 1.260057 1.92 0.055 0.9783309 6.717876

AKI | 3.689404 1.501325 3.21 0.001 1.661796 8.190959

Bacterial infection | 3.065492 1.289963 2.66 0.008 1.343734 6.993377

Child-Pugh score | 1.742471 .2305884 4.20 0.000 1.344381 2.25844

Family history of thrombosis | 3.683918 2.344553 2.05 0.040 1.058231 12.82447

Reduced mobility | 5.830839 2.541037 4.05 0.000 2.481896 13.69867

CRP/10 | 1.186353 .0716192 2.83 0.005 1.053968 1.335366

_cons | .0000439 .0000737 -5.99 0.000 1.64e-06 .0011741

-------------------------------------------------------------------------------------------------------------------

Note: _cons estimates baseline odds.

*lroc*

AUROC = 0.8822

Notably, we would want to highlight that multivariable logistic models can serve a dual purpose. Firstly, they address the etiology of a disease (or causal inference), a process often defined as “explanatory modeling”, which uses statistical models to test causal explanations. In such a model, all clinically relevant variables that may have a causal role (i.e., independent variables) must be included. In these models, the selection of variables is crucial, whereas the prognostic performance is relatively less significant. The second purpose is the development of a clinical prediction model. The variables included in predictive models must not have an inferential aim. However, variables with a p-value above 0.05 can be selected in such a model since they may be important for improving the prediction ability of the model. In other words, the model's prognostic performance is what matters most, not the statistical significance of the variables included in the model. Since, in this study, we aimed to develop a predictive model, the variable "male gender" was included in the final model, as its inclusion improved the model's prognostic ability.

***5. ****GENERATE RISK PREDICTIONS ON THE PROBABILITY SCALE (0-100%)******

*The final model described above was used to generate VTE risk predictions. We input the predictors of new patients into the logistic function and obtained a value between 0 and 1. This corresponds to a percentage scale of 0-100% risk. This process was the same as calculating the propensity score of 687 enrolled patients (i.e., individual risk of developing VTE). The final equation obtained by our model was:

*Logistic group sex, AKI, bacterial infection, Child-Pugh score, history of thrombosis, reduced mobility, CRP/10, coef*

Logistic regression Number of obs = 687

LR chi2(7) = 81.87

Prob > chi2 = 0.0000

Log likelihood = -94.410065 Pseudo R2 = 0.3025

-----------------------------------------------------------------------------------------------------------------

Variable | Coefficient Std. err. z P>|z| [95% conf. interval]

-------------+--------------------------------------------------------------------------------------------------

Sex | 0.9414324 .4915089 1.92 0.055  **-.0219074** **1.904772**

AKI | 1.305465 .4069289 3.21 0.001 .5078991 2.103031

Bacterial infection | 1.120208 .4208014 2.66 0.008 .2954525 1.944964

Child-Pugh score | .555304 .1323342 4.20 0.000 .2959338 .8146742

Family history of thrombosis | 1.303977 .6364292 2.05 0.040 .0565987 2.551355

Reduced mobility | 1.763161 .4357928 4.05 0.000 .9090227 2.617299

CRP/10 | .1708836 .0603692 2.83 0.005 .0525621 .2892051

_cons | -11.91543 1.723445 -6.91 0.000 -15.29332 -8.537538

----------------------------------------------------------------------------------------------------------------

***E = -10.03 + 0.94 [Male sex] + 1.31 [AKI] + 1.12 [bacterial infection] + 0.56*Child-Pugh score + 1.30 [VTE family history] + 1.76 [Reduced mobility] + 0.17 * PCR/10**

***Here, the brackets "[ ]" equal one if the patient has the characteristic in question (i.e., if the patient is male, has AKI, has a bacterial infection, has VTE family history, has reduced mobility), and 0 otherwise.**

***Predicted VTE risk: 1 / (1+ Exp (-E))**

***Simplified score: E + 10**

*In the figure below, we plotted enrolled patients' predicted VTE risk over the simplified score derived from our final equation (E).


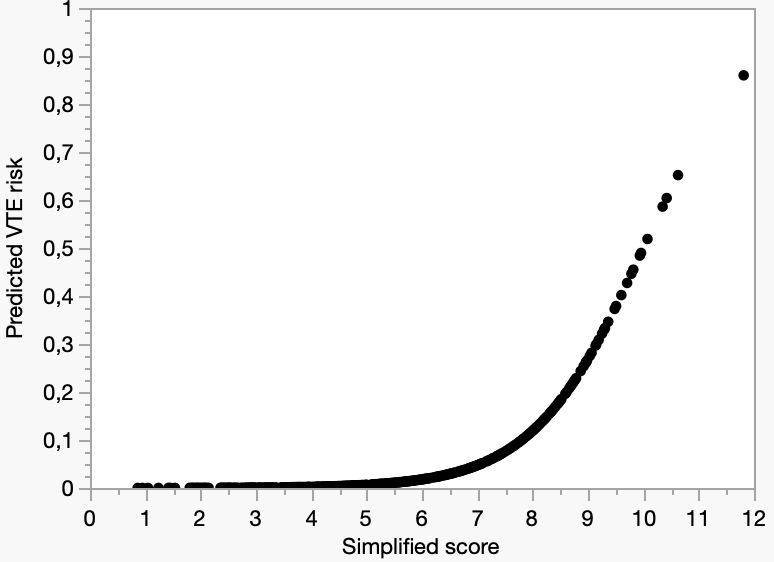


***6. ****EVALUATE THE MODEL'S PERFORMANCE REGARDING DISCRIMINATION, CALIBRATION, AND CLINICAL UTILITY******

*****DISCRIMINATION*****

*Our model's discrimination ability was evaluated by measuring the area under (AU) the receiver-operating characteristic (ROC) curve.

*Our model's calculated AUROC was 0.8822. We compared its discrimination ability with that of the Padua score.


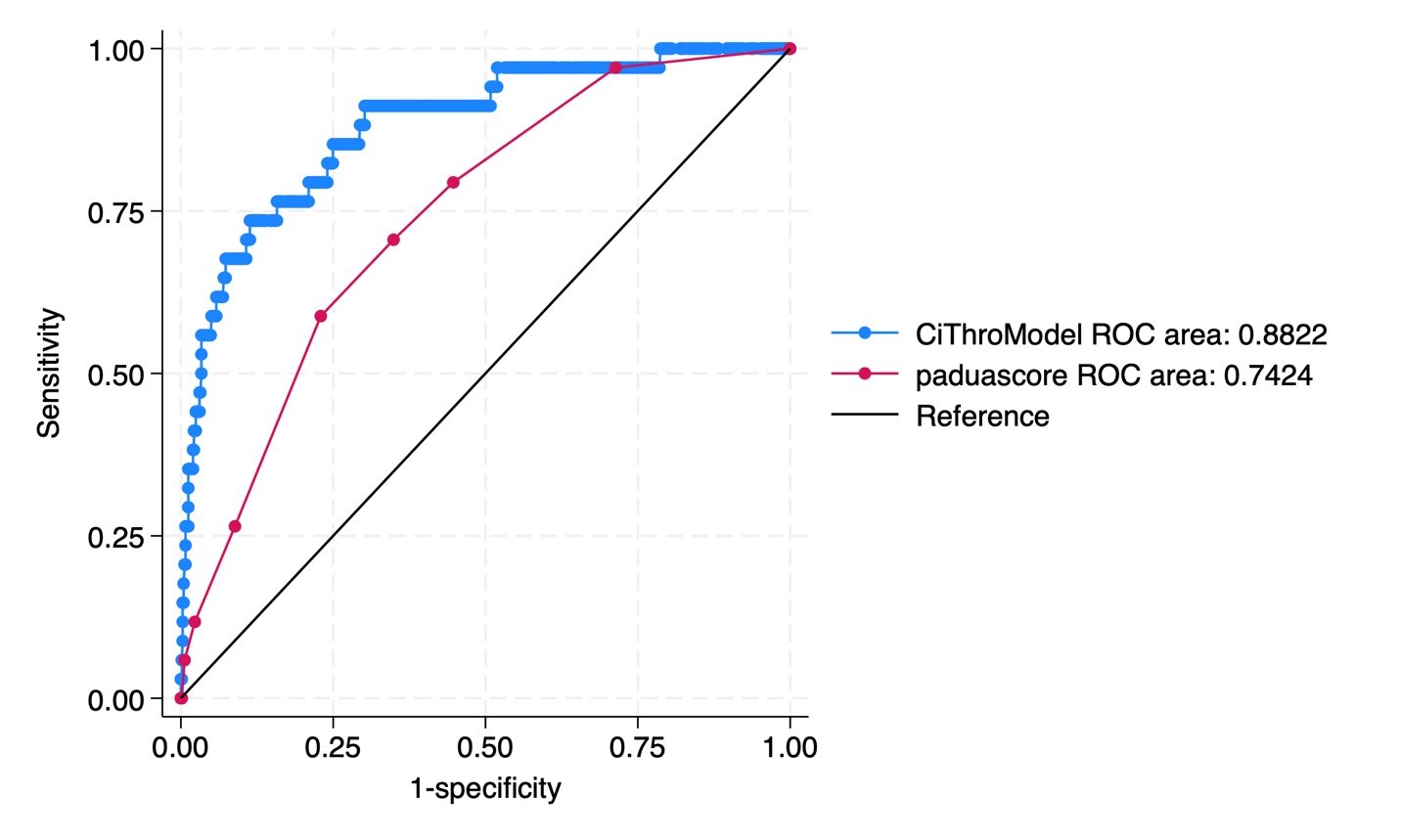


The calculated AUROC for the Padua score was 0.7424

The delta AUROC was 14% (p<0.001)

ROC Asymptotic normal

Obs area Std. err. [95% conf. interval]

-------------------------------------------------------------------------

phat 687 0.8822 0.0317 0.82003 0.94427

Padua score 687 0.7424 0.0392 0.66561 0.81912

-------------------------------------------------------------------------

H0: area(phat) = area (Padua score)

chi2(1) = 11.52 Prob>chi2 = 0.0007

*****CALIBRATION*****

* To test the calibration of our model, we performed two analyses.

***a) Calibration belt**

*Calibration belt group CiThroModel, devel ("internal")*


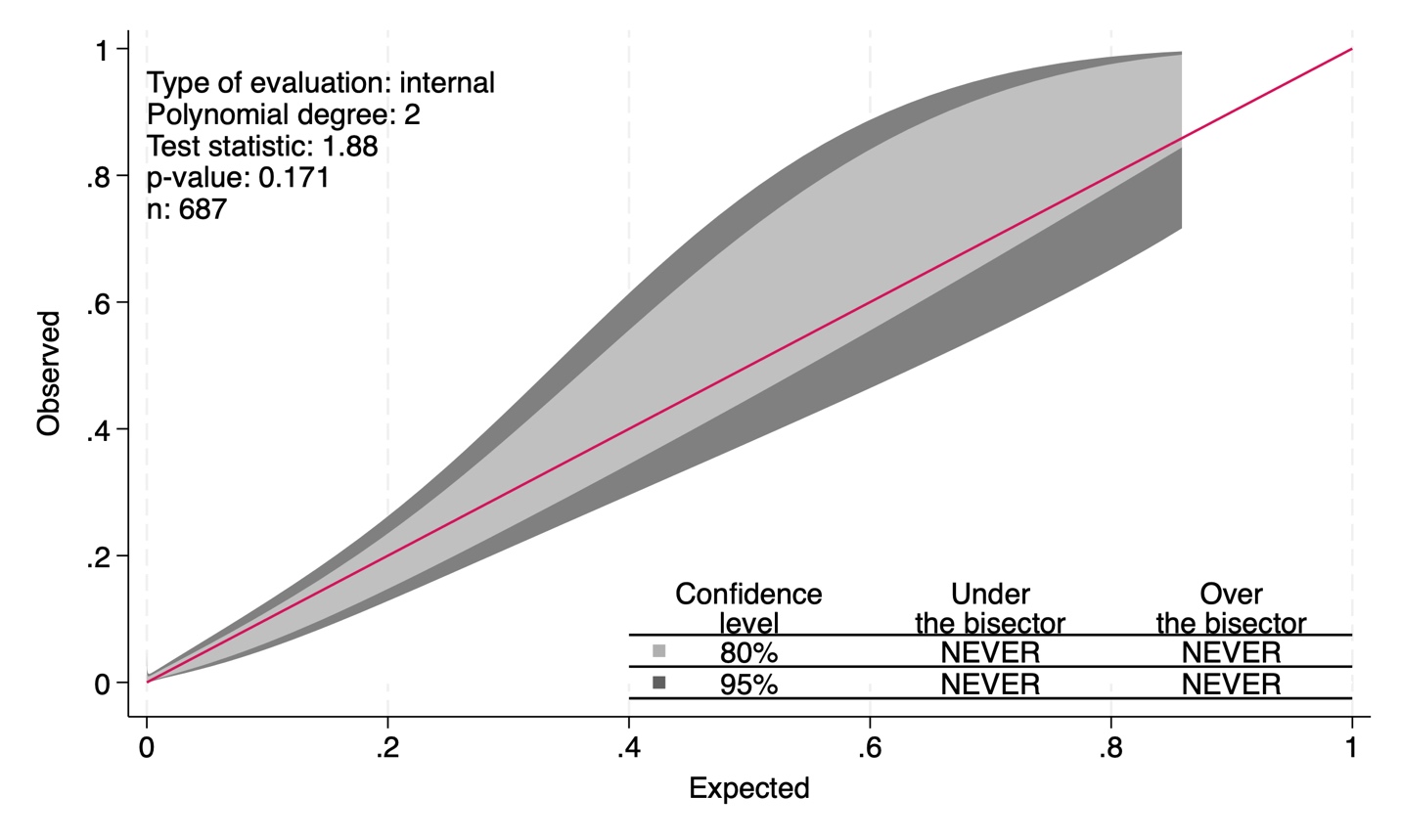


*The calibration belt above reports the value of the statistic (1.88) and the p-value (0.171) of the test. These results suggest that the hypothesis of good calibration is not rejected (at the classically adopted 0.05 level). Similar conclusions can be drawn from interpreting the produced plot, as reported in the figure. We note that both the 80% and 95% calibration belts encompass the bisector over the whole range of the predicted probabilities. This suggests that the model's predictions are consistent with the observed rate in the developmental sample (the model's internal calibration is acceptable).

***b) Calibration plot**


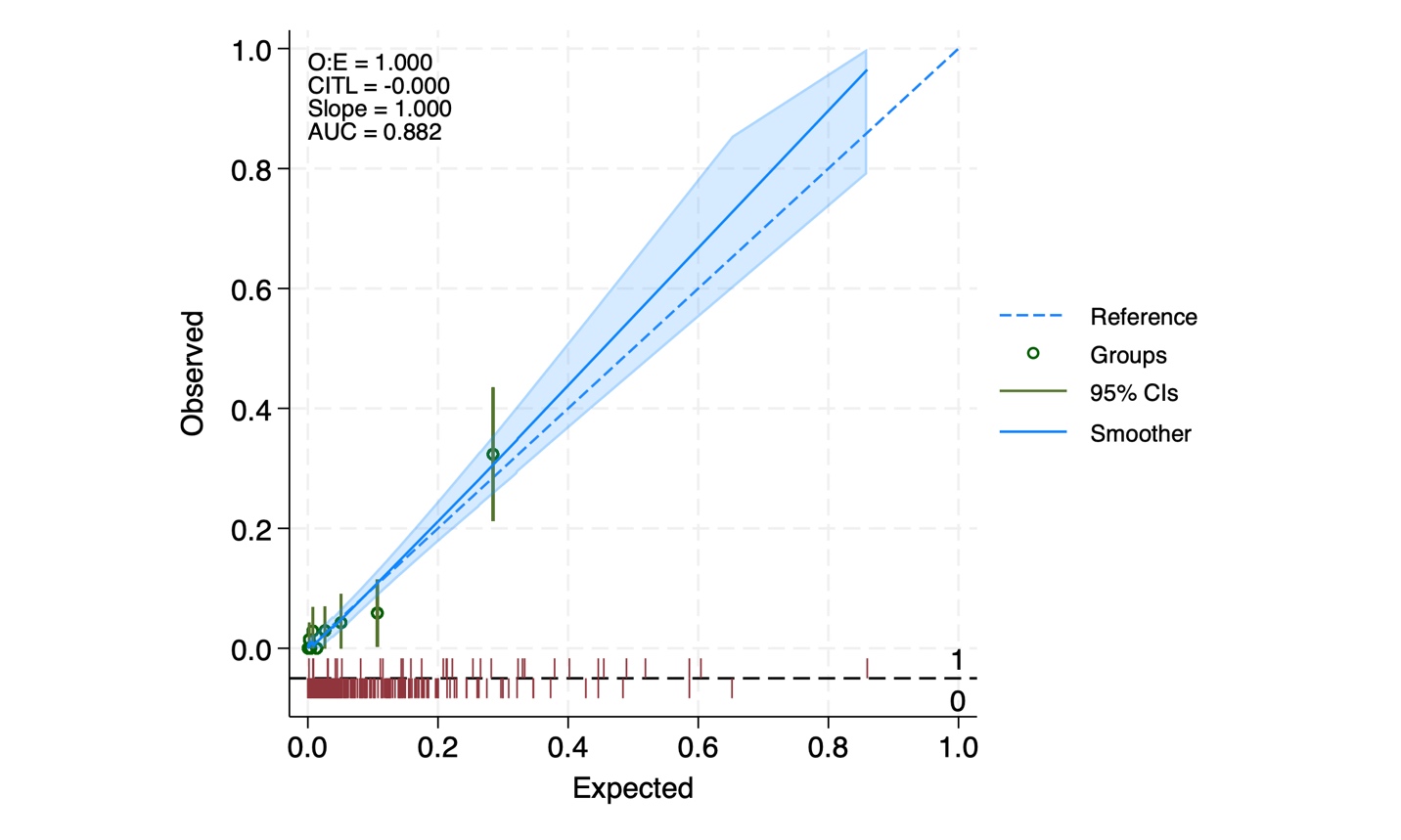


*We also constructed a calibration plot. The observed/expected ratio equal to 1 indicates that the average predicted risk corresponds to the proportion of observed events. The estimated slope coefficient was also 1. This suggests no over- (slope less than 1) or underfitting (slope greater than 1) of our model.

*****CLINICAL UTILITY*****

**Decision curve analysis (DCA) **

*tutorial: https://mskcc-epi-bio.github.io/decisioncurveanalysis/dca-tutorial.html

*dca group CiThroModel padua, smooth xstop(0.6) xlabel(0(0.05)0.6) lcolor(black gs8 black) lpattern(solid solid dash) title("Decision Curve Analysis", size(4) color(red)) scheme(stcolor_alt)*

*see Figure 5 in the main text

**"A decision needs to be made for the patients based on their predicted risk. This means using a threshold or cut-off for risk. We performed a net benefit (decision curve) analysis. In our model, "the intervention" we wish to apply in VTE high-risk patients is anticoagulation therapy. We can assume a benefit to correctly treating those with VTE (true positive) and some harm to unnecessarily treating those without VTE (false positive). The model's number of TPs and FPs depends on the chosen risk threshold (p), as only those above it receive treatment. The total net benefit (NB) of treating the resulting number of TPs is then compared against the total harm of treating the resulting FPs. NB (p) = TP/N – (p/(1-p))*FP/N, Where N is the total number of patients in the dataset. The threshold probability we choose determines the acceptable trade-off between benefit and harm. If we chose, for example, 20% as the threshold, we would be willing to treat five people (one correctly and four unnecessarily)"(1).*


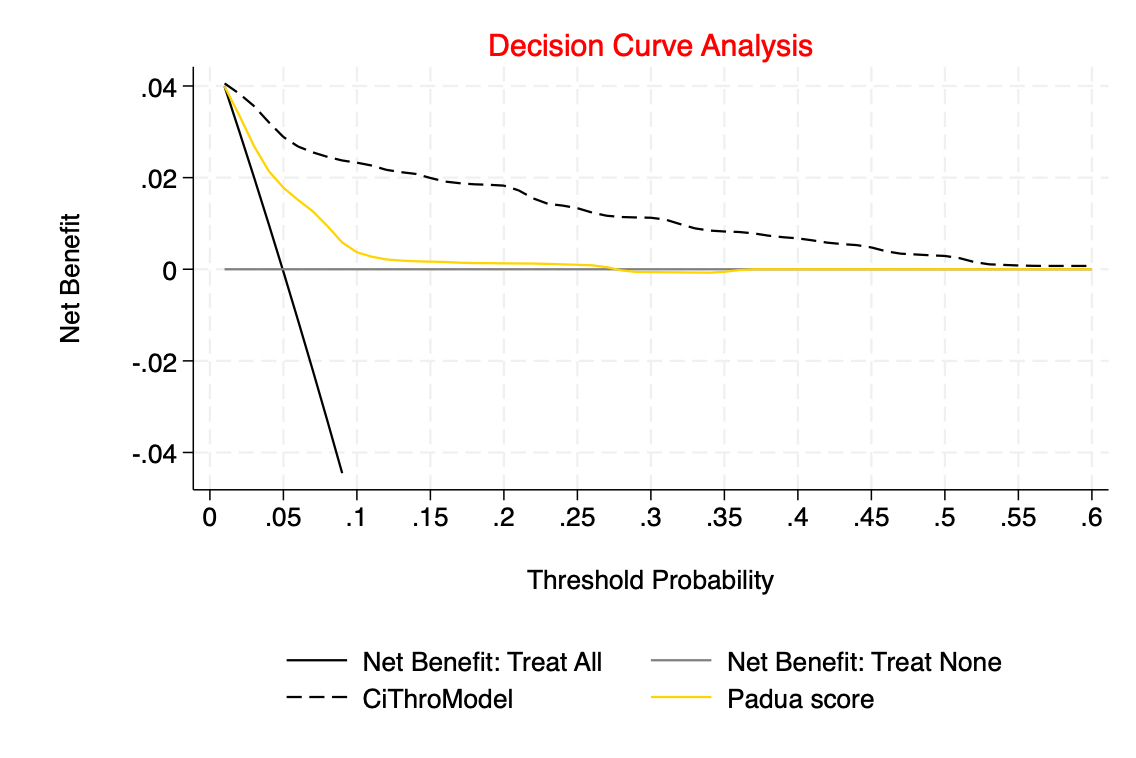


* The figure shows the net benefit analysis (i.e., decision curve). This analysis highlights the clear superiority of the CiThroModel compared to the Padua score in terms of net benefit (i.e., the relative benefits and harms of VTE prophylaxis at different thresholds of predicted VTE probability). For instance, at a given threshold probability of 0.10, the figure shows a difference in net benefit of 0.020 between the CiThroModel and the Padua score. This difference could be interpreted as “using the CiThroModel instead of the Padua score to decide VTE prophylaxis increases the number of VTE detected (true positives) by 20 per 1000 patients, without changing the number of unnecessary treatments (false positives).”

***7. ****VALIDATE THE MODEL USING BOOTSTRAPPING TO CORRECT FOR THE APPARENT OPTIMISM IN PERFORMANCE******

*Logistic variable sex, AKI, bacterial infections, Child-Pugh score, family history of thrombosis, reduced mobility, CRP/10*

Apparent performance

------------------------------------------------------------

[95% Conf. Interval]

Overall:

Brier scaled (%) = 22.8

Discrimination:

C-Statistic = 0.882 0.820 0.944

Calibration:

E:O ratio = 1.000

CITL = -0.000 -0.384 0.384

Slope = 1.000 0.714 1.286

------------------------------------------------------------

Bootstrap performance (Optimism adjusted)

Number of replications: 50

------------------------------------------------------------

[Bootstrap 95% CI]

Overall:

Brier scaled (%) = 16.7

Discrimination:

C-Statistic = 0.862 0.780 0.939

Calibration:

E:O ratio = 0.980 0.644 1.356

CITL = 0.062 -0.387 0.659

Slope = 0.878 0.483 1.281

------------------------------------------------------------

Shrinkage factors

------------------------------------------------------------

Heuristic Shrinkage = 0.915

Bootstrap shrinkage = 0.878

------------------------------------------------------------

***After bootstrapping, the adjusted model maintained an optimal discrimination ability (C-Statistic of 0.862) and calibration (E:O ratio = 0.980, CITL = 0.062, Slope = 0.878).**

* Adjusted calibration plot


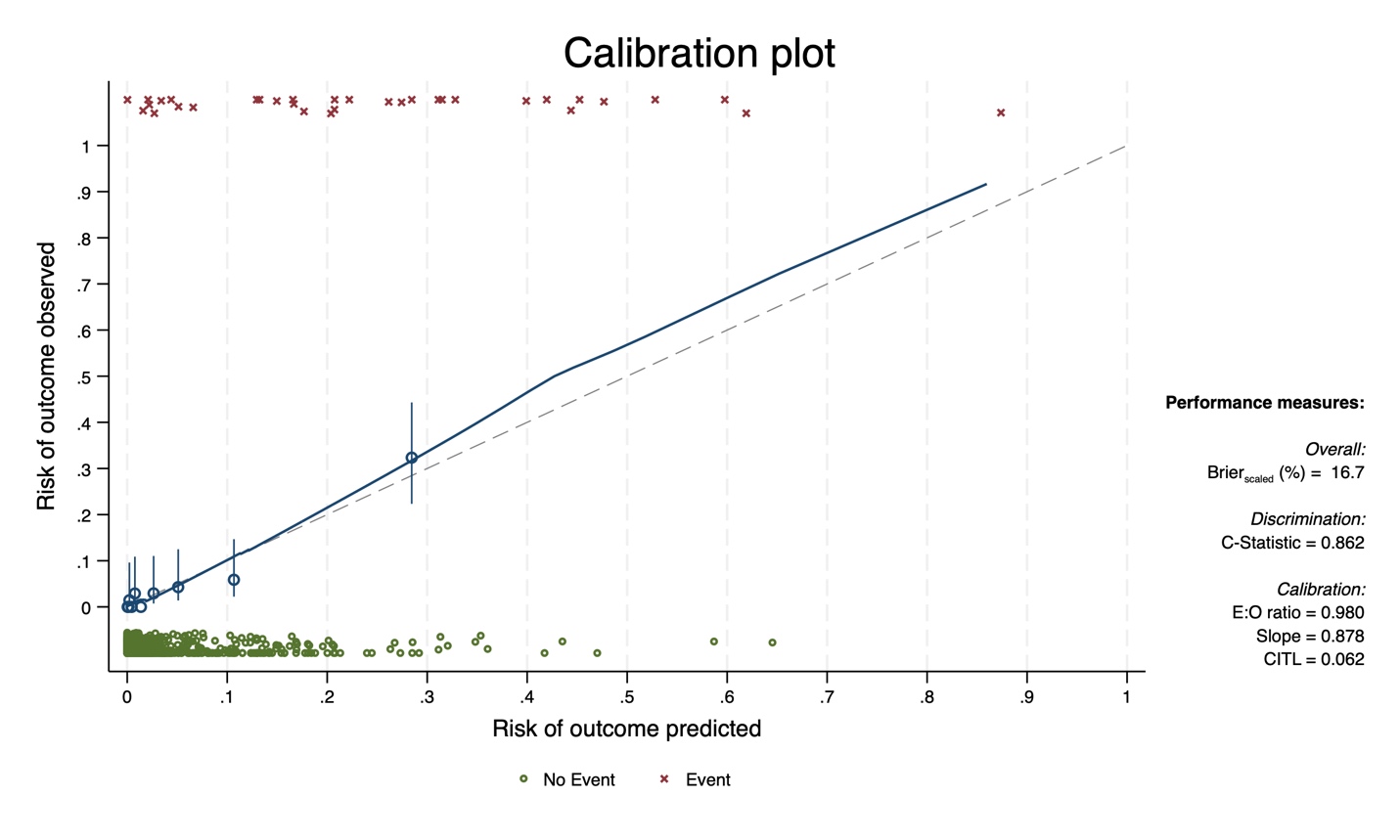


*Logistic variable sex, AKI, bacterial infections, Child-Pugh score, family history of thrombosis, reduced mobility, CRP/10*

*bsvalidation, rseed(123) graph*

*** Adjusted OR**

Logistic variable sex, AKI, bacterial infections, Child-Pugh score, family history of thrombosis, reduced mobility, CRP/10

*bsvalidation, rseed(123) reps(100) pr(0.1) adjust(bootstrap) eform*

Model adjusted by bootstrap shrinkage

-----------------------------------------------------------------------------------------------------------------

Variable | Odds Ratio Std. err. z P>|z| [95% conf. interval]

-------------+--------------------------------------------------------------------------------------------------

Sex | 2.270472 0.9719986 1.92 0.055 0.9810996 5.254351

AKI | 3.117589 1.104983 3.21 0.001 1.556407 6.244743

Bacterial infection | 2.653027 .9723821 2.66 0.008 1.293484 5.441546

Child-Pugh score | 1.622016 .1869585 4.20 0.000 1.294026 2.03314

Family history of thrombosis | 3.113551 1.725934 2.05 0.040 1.050533 9.227889

Reduced mobility | 4.644636 1.76299 4.05 0.000 2.207275 9.77343

CRP/10 | 1.160487 .0610203 2.83 0.005 1.046846 1.286464

_cons | .0001237 .0000237 -46.98 0.000 .000085 .00018

-----------------------------------------------------------------------------------------------------------------

*** Adjusted coefficients**

*Logistic variable sex, AKI, bacterial infections, Child-Pugh score, family history of thrombosis, reduced mobility, CRP/10*

*bsvalidation, rseed(123) reps(100) pr(0.1) adjust(bootstrap)*

------------------------------------------------------------------------------------------------------------------

Model adjusted by bootstrap shrinkage

------------------------------------------------------------------------------------------------------------------

Variable | Coefficient Std. err. z P>|z| [95% conf. interval]

-------------+---------------------------------------------------------------------------------------------------

Sex | .8199876 .4281042 -1.92 0.055 -1.659056 .0190813

AKI | 1.13706 .3544351 3.21 0.001 .4423801 1.83174

Bacterial infection | .9757012 .366518 2.66 0.008 .2573391 1.694063

Child-Pugh score | .4836698 .1152631 4.20 0.000 .2577583 .7095812

Family history of thrombosis | 1.135764 .5543298 2.05 0.040 .0492974 2.22223

Reduced mobility | 1.535713 .3795755 4.05 0.000 .7917588 2.279667

CRP/10 | .1488396 .0525816 2.83 0.005 .0457816 .2518977

_cons | -8.997819 .1915222 -46.98 0.000 -9.373196 -8.622443

-------------------------------------------------------------------------------------------------------------------

**ADJUSTED (after bootstrapping) CiThroModel**

***We create the adjusted (final) CiThroModel with the new coefficients**

***E = -9.00 + 0.82 [Male sex] + 1.14 [AKI] + 0.98 [Infection] + 0.48*Child Pugh score + 1.14 [VTE family history] + 1.54 [Reduced mobility] + 0.15 * PCR/10.**

***Here, the brackets "[ ]" equal 1 if the patient has the characteristic in question (i.e., if the patient is male, has AKI, has an infection, has VTE family history, has reduced mobility), and 0 otherwise.**

***Predicted VTE risk: 1 / (1+ Exp (-E))**

**References**

1. Strandberg R, Jepsen P, Hagstrom H. Developing and validating clinical prediction models in hepatology - An overview for clinicians. J Hepatol. 2024.

2. Blanche PF, Holt A, Scheike T. On logistic regression with right censored data, with or without competing risks, and its use for estimating treatment effects. Lifetime Data Anal. 2023;29(2):441-82.

3. Coemans M, Verbeke G, Dohler B, Susal C, Naesens M. Bias by censoring for competing events in survival analysis. BMJ. 2022;378:e071349.

4. Poguntke I, Schumacher M, Beyersmann J, Wolkewitz M. Simulation shows undesirable results for competing risks analysis with time-dependent covariates for clinical outcomes. BMC Med Res Methodol. 2018;18(1):79.

5. Zanetto A, Campello E, Spiezia L, Burra P, Simioni P, Russo FP. Cancer-Associated Thrombosis in Cirrhotic Patients with Hepatocellular Carcinoma. Cancers (Basel). 2018;10(11).
